# Diffusion-Weighted MRI as a Quantitative Imaging Biomarker in Colon Tumors

**DOI:** 10.3390/cancers16010144

**Published:** 2023-12-27

**Authors:** Peter Obel Otto, Martina Kastrup Loft, Søren Rafael Rafaelsen, Malene Roland Vils Pedersen

**Affiliations:** 1 Department of Radiology, University Hospital of Southern Denmark, Beriderbakken 4, 7100 Vejle, Denmark; 2 Danish Colorectal Cancer Center South, Vejle Hospital, 7100 Vejle, Denmark; 3 Department of Regional Health Research, University of Southern Denmark, Campusvej 55, 5230 Odense, Denmark

**Keywords:** colon cancer, adenoma, diffusion weighted imaging, MRI, DWI, ADC, mucinous

## Abstract

**Simple Summary:**

Adenomas may be mistaken for a malignant tumor during colonoscopy. Patients with confirmed colon tumors during colonoscopy underwent a Magnetic Resonance imaging (MRI) investigation using a 3.0 Tesla unit. We aimed to assess the usefulness of quantitative, diffusion-weighted MRI (DW-MRI) in colon tumors, in which accurate staging is becoming increasingly important, with new and more personalized treatment options. The endpoint was the surgically resected tumor specimens the mucinous colon tumors was classified based on T2 weighted images. We observed a significantly higher degree of restricted diffusion in colon carcinomas compared to adenomas. However, this difference was less pronounced in cases of mucinous-producing tumors.

**Abstract:**

Purpose: To assess the use of quantitative diffusion-weighted MRI (DW-MRI) as a diagnostic imaging biomarker in differentiating between benign colon adenoma, early, and advanced cancer of the colon, as well as predicting lymph node involvement, and finally comparing mucinous-producing colon cancer with adenomas and non-mucinous colon cancer. Method: Patients with a confirmed tumor on colonoscopy were eligible for inclusion in this study. Using a 3.0 Tesla MRI machine, the main tumor mean apparent diffusion coefficient (mADC) was obtained. Surgically resected tumor specimens served as an endpoint, except in mucinous colon cancers, which were classified based on T2 images. Results: A total of 152 patients were included in the study population. The mean age was 71 years. A statistically significant mADC mean difference of −282 × 10^−6^ mm^2^/s [−419–−144 95% CI, *p* < 0.001] was found between colon adenomas and early colon cancer, with an AUC of 0.80 [0.68–0.93 95% CI] and an optimal cut off value of 1018 × 10^−6^ mm^2^/s. Only a small statistically significant difference (*p* = 0.039) in mADC was found between benign tumors and mucinous colon cancer. We found no statistical difference in mADC mean values between early and advanced colon cancer, and between colon cancer with and without lymph node involvement. Conclusion: Quantitative DW-MRI is potentially useful for determining whether a colonic tumor is benign or malignant. Mucinous colon cancer shows less diffusion restriction when compared to non-mucinous colon cancer, a potential pitfall.

## 1. Introduction

The accurate preoperative staging of colon cancer, and differentiating between adenoma and early T stages of adenocarcinoma, continues to challenge clinicians. The recommended modality and gold standard for staging colon cancer is Contrast-Enhanced Computed Tomography (CECT) of the thorax and abdomen [1]. Magnetic Resonance Imaging (MRI) can be an improvement compared to CECT, however, lymph node involvement remains difficult to ascertain [1,2,3,4]. Furthermore, MRI has the potential to determine if a tumor has invaded beyond the layers of the colon wall or nearby structures. MRI is routinely used in assessing liver metastases.

Neoadjuvant therapy has had a positive impact in treating advanced rectal cancer. Accurately staging rectal tumors is imperative when planning an individualized treatment strategy [5]. The Foxtrot study, a large ongoing trial, evaluates the potential impact of neoadjuvant therapy in patients with colon cancer [6]. With new potential treatment strategies, the importance of accurate staging will increase further [7]. Combining neoadjuvant therapy with immunotherapy has shown promising outcomes, and microsatellite instability can enhance tumor immunogenicity [8].

Overtreatment of colorectal tumors is a well-known risk. Patients with non-malignant adenomas may undergo partial colectomy, when polypectomy or mucosectomy would be less costly, less demanding for the patient, and have a lower rate of complications [9,10]. On the other hand, colonic tumors with an endoscopically benign appearance, commonly polypoid, are often removed by polypectomy or mucosectomy. The histopathological report of these tumors sometimes reveals the presence of malignancy. This poses a dilemma for clinicians, as potentially undertreated patients may require radical surgery and follow-up [11]. Diffusion Weighted Imaging (DWI) is a functional MRI technique that visualizes the random microscopic motion of mainly water molecules within the body, both intra- and extracellularly. Malignant tissue typically has increased cellular density compared to healthy tissue and exhibits restricted diffusion, in contrast to surrounding healthy tissue, on DWI [12]. The apparent diffusion coefficient (ADC) is a quantitative measurement of diffusion restriction. Visualized as an ADC map with each voxel having a calculated diffusion rate expressed in the unit mm^2^/s [12,13]. The ADC map derived from DWI quantifies diffusion restriction, potentially allowing for the evaluation of tissue as malignant or benign. The ADC map can also visually indicate whether there is diffusion restriction or not. Dark areas represent diffusion restriction, while bright areas on the ADC map indicate the absence of diffusion restriction.

In rectal cancer, DWI is used in follow-up to differentiate between scar tissue and residual cancer tissue after neoadjuvant therapy [14,15,16,17]. DWI is currently recommended by the European Society of Gastrointestinal and Abdominal Radiology (ESGAR) to be used only qualitatively and not quantitatively, because of a lack of standardization between different MRI systems and imaging protocols [5]. To the best of our knowledge, only one other study with eight adenomas and seven carcinomas has used whole-body DWI to attempt to differentiate colon adenomas from carcinomas [18].

This study aims to assess the potential usefulness of the main tumor mean ADC (mADC) as a quantitative imaging biomarker for differentiating between benign adenoma, early-stage, and advanced cancer of the colon on a larger scale and determining lymph node involvement. Additionally, to investigate variations in tumor mean mADC between adenomas, mucinous, and non-mucinous adenocarcinomas of the colon.

## 2. Materials and Methods

### 2.1. Patients

This study included a population of 152, of which 72 were female. Median and mean age were both 71 years, ranging between 39 and 92 years. The inclusion was carried out over a two-year period from 2018 to 2020.

**Figure 1:**
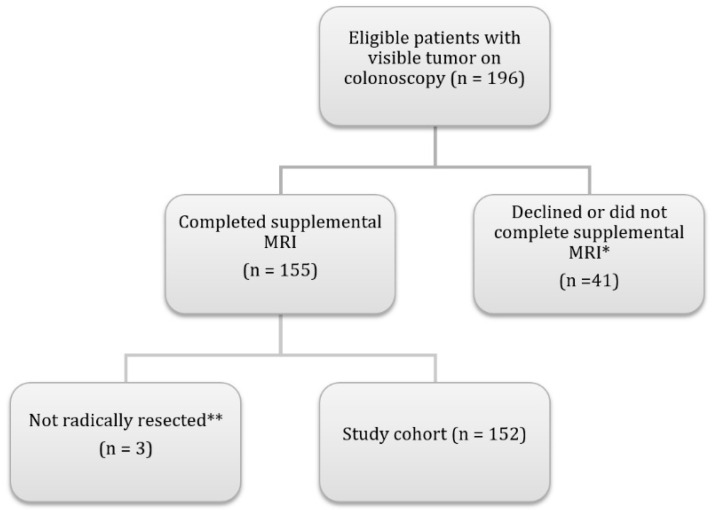
Flow chart demonstrating the inclusion of patients in the study. * Reasons were, declined to participate in study (*n* = 16), emergency surgery (*n* = 13), neoadjuvant therapy (*n* = 8), claustrophobia (*n* = 3), and pacemaker (*n* = 1). ** These three patients had a benign biopsy result, with no resection performed. Abbreviations: n, number of patients.

Data used in this study were derived from a prospectively included cohort [3], including patients with a visible tumor on colonoscopy. Patients underwent a standardized CECT scan and a supplementary MRI of the abdomen and pelvis. A flow chart demonstrating inclusion and exclusion criteria is presented in Figure 1.

All included patients had radical resection performed within 30 days of the MRI exam, and none received neoadjuvant therapy. Inclusion criteria were >18 years old and suspicion has been raised about a colon tumor or suspicious looking polyp from colonoscopy. Exclusion criteria were if the patient could not undergo an MRI due to, e.g., claustrophobia, pacemaker or drug pumps, or prior radiotherapy/chemotherapy. 

All included patients underwent the MRI exam using a 3.0 Tesla Philips Ingenia MRI machine (Philips Medical Systems, Best, The Netherlands) with a bore diameter of 70 cm. No patient preparation prior to MRI was required, and no contrast medium or spasmolytic agent was administered. Patients were scanned in a supine position, using a posterior coil embedded in the flatbed of the MRI machine and a body coil placed anteriorly covering the pelvis and the abdomen. 

### 2.2. Imaging

The MRI protocol consisted of two T2-weighted and one DWI (b0-b800) sequence covering the entire abdominal and pelvic region. Furthermore, an additional T2-weighted followed by a DWI (b0-b1000) sequence covering the tumor, and angulated perpendicular to the colon tumor, planned and angled with assistance from an experienced gastrointestinal radiologist. A respiration trigger was used for all sequences to reduce blurring from movement due to breathing. All T2-weighted sequences utilized vendor proprietary ‘MultiVane XD’, a type of oversampling, further reducing blurring from movement, including peristaltic bowel movements, and thereby improving image quality [19]. Details on the MRI sequences are shown in Table 1.

The T2 and the DWI (b0-b800) images were primarily used for visualizing and localizing tumors, as well as any potential tumor spread. ADC grayscale image maps were automatically generated from the angulated DWI b0 and b1000 images. The ADC maps contain quantitative ADC values for each voxel. 

The mADC values were determined by placing a free-hand region of interest (ROI) covering the area of the tumor with the lowest signal intensity on the ADC map, carefully avoiding artefacts from bowel air and partial volume voxels. This ROI placed on the ADC map represents the part of the tumor with the highest signal intensity on the corresponding b1000 image slice, thus creating a sample ROI of the most diffusion-restricted area of the tumor on a single slice. The image slice was chosen based on tumor visibility and tumor area size, as well as signal intensity on the b1000 images. Tumor classification followed the UICC AJCC 8th edition TNM classifications system [20] and was performed on the T2 angulated images. An experienced gastrointestinal radiologist, who was blinded to the pathology results, assessed tumor stage, in the following indicated by the prefix ‘mr’, and obtained mADC values. ADC is provided in the unit: 10^−6^ mm^2^/s. 

### 2.3. Endpoint

The histopathological TN classification, indicated by the ‘p’ prefix, served as an end-point. The tumors were divided into three groups depending on pT stage: pathology-proven adenomas (pAdenoma); early cancer, defined as pT1 or pT2 tumors (pT1–pT2); and advanced cancer, defined as pT3 or pT4 stage (pT3–pT4). Patients with colon cancer were also divided into two groups depending on lymph node involvement: pN0 and pN+. Finally, adenocarcinomas were grouped into non-mucinous cancer (mrNMCC) and mucinous cancer (mrMCC), based on the determination of the reporting radiologist.

### 2.4. Statistics

IBM SPSS Statistics software version 27.0.0.0 (IBM Corporation, Armonk, NY, USA) was used for statistical tests. *p* values ≤ 0.05 were considered statistically significant. 

Cases of extreme outliers were excluded prior to statistical calculations. All data were checked for normal distribution using histograms, which were visually compared to normal probability curves, Q–Q plot assessment, and the Shapiro–Wilks test. The independent samples’ *t*-tests and 95% confidence intervals were used to compare the mADC group means. Receiver Operator Characteristics (ROC) analysis was performed between relevant groups to calculate the area under the curve (AUC) and the optimal cut-off values, based on the shortest distance from the curve to the upper left corner, representing the highest combined sensitivity and specificity.

### 2.5. Ethics

This study was approved by the Regional Committees on Health Research Ethics for Southern Denmark (S-20180078). Data processing was approved by the Region of Southern Denmark (22-35801). Written and oral consent was obtained from all patients prior to the supplementary MRI exam. 

## 3. Results

**Figure 2:**
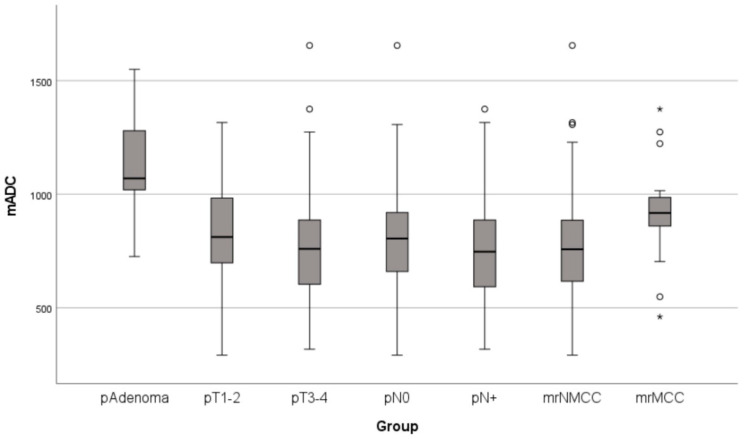
Box plot showing median, quartiles, and outliers for each group. Abbreviations: mADC, main tumor mean apparent diffusion coefficient in 10^−6^ mm^2^/s; mrNMCC, non-mucinous colon cancer determined using magnetic resonance imaging; mrMCC, mucinous colon cancer determined using magnetic resonance imaging. * = outliers.

Tumor location and characteristics are shown in Table 2. Figure 2 shows a box plot detailing the median, quartiles, and outliers for each group. All benign tumors were adenomas. Two extreme outliers, a pT3 tumor with an ADC value of 2168 × 10^−6^ mm^2^/s and a pT2 tumor with an ADC value 227 × 10^−6^ mm^2^/s, were excluded.

**Figure 3:**
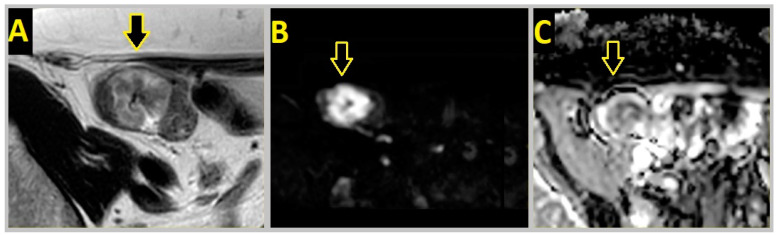
A 78-year-old woman with a tumor (marked by a yellow arrow) in the ascending colon as identified with colonoscopy. (**A**): Axial T2-weighted MRI image of the polyp-shaped tumor. (**B**): b1000 MRI image showed a strong signal. (**C**): Apparent diffusion coefficient map. The tumor’s mean apparent diffusion coefficient (delineation not shown) was 1110 × 10^−6^ mm^2^/s.

**Figure 4:**
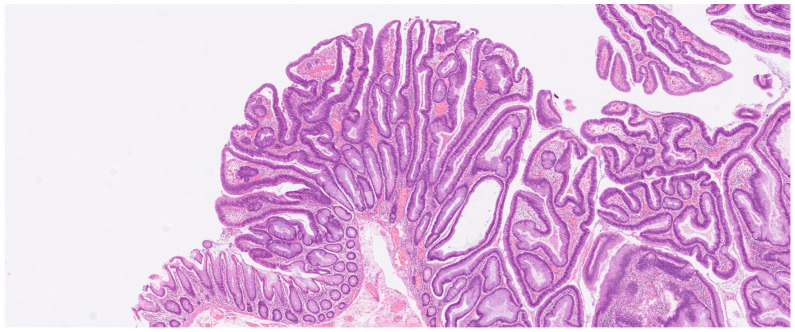
Microscopic image of 10× magnification showing resected tumor from the same patient as Figure 3. The patient underwent laparoscopic robotic-assisted right-sided hemicolectomy. Histopathology showed a tubolovillous adenoma and no malignancy.

Figure 3 and Figure 4 show MRI images and a microscopic image of a resected tumor, respectively, from the same patient.

Although not shown in Table 3, there was a statistical difference in mADC means between pAdenoma and pT3–pT4, with a mean difference of −346 × 10^−6^ mm^2^/s, with a 95% confidence interval of −234 × 10^−6^ mm^2^/s to −459 × 10^−6^ mm^2^/s and *p* < 0.001. Overall, it can be difficult to distinguish using mADC values as there is little variance, but the mADC can provide a guideline for future explorations. 

The group mean mADC and comparison between groups are shown in Table 3.

**Figure 5:**
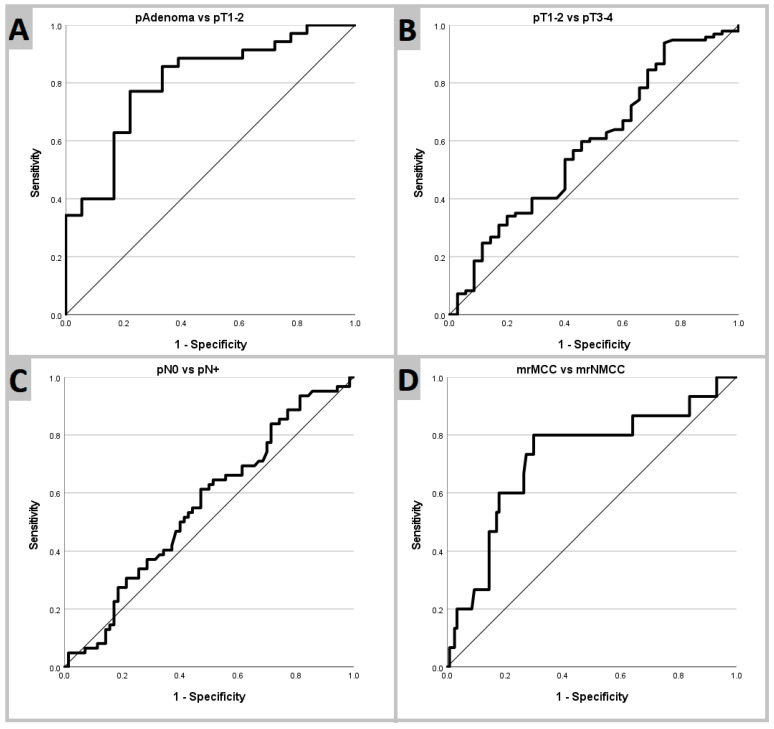
Receiver operating characteristic curves. (**A**): pAdenoma vs. pT1–pT2. (**B**): pT1–pT2 vs. pT3–pT4. (**C**): pN0 vs. pN+. (**D**): mrMCC vs. mrNMCC. Abbreviations: vs, versus; mrNMCC, non-mucinous colon cancer determined using magnetic resonance imaging; mrMCC, mucinous colon cancer determined using magnetic resonance imaging.

Receiver operating characteristic analysis and curves are shown in Table 4 and Figure 5, respectively.

## 4. Discussion

We found a statistically significant difference in mADC values between adenomas and early colon cancers. The AUC in differentiating adenomas from pT1–pT2 was 0.80. This is in agreement with a study on rectal polyps [21]. Other rectal MRI studies have shown similar results [15,22,23], but the literature is scarce on colonic tumors. We found no statistical difference in mADC values between N0 and N+ groups, and pT1–pT2 and pT3–pT4 groups.

Methods for obtaining ADC differ between studies. Arponent et al. [24] compared small sub-segment ROIs, delineating the brightest part on b1000, to whole-lesion ROI, covering the entire tumor, in breast tumor analysis. Cut-off values differed significantly, and the study concluded that ADC values from small sub-segment ROIs more accurately represented the most aggressive tissue component, showing a higher association with prognostic factors [24]. In this study, the method used for obtaining an ADC value was tracing the part of the tumor that was brightest on b1000 by free hand. This method appears to be a strength when the goal is to determine whether a tumor is benign or cancerous, as the mean mADC value difference between these two groups was strongly statistically significant, with no overlap in 95% confidence intervals. However, this method seems unable to distinguish between early and advanced cancer and to determine lymph node metastasis. A similar study by Nerad et al. [25], who used a whole tumor volume mean ADC value approach, showed a greater ability to differentiate between early and advanced cancer, as well as to predict lymph node involvement [25]. 

Mucinous-producing tumors have a higher mean mADC compared to non-mucinous tumors, confirmed by Çolakoğlu et al. in a similar study [26]. The higher mean mADC values are caused by the presence of extracellular mucin, a viscous liquid, which increases diffusion [26]. This is a potential pitfall when assessing tumors quantitatively by mADC, as mucinous colon cancers have a smaller mADC difference than non-mucinous cancers when compared with benign adenomas. Mucinous tumors appear bright on T2-weighted images, assisting in qualitative interpretation [26].

As the field of diagnostic MRI continues to evolve, with image quality improving and acquisition times decreasing, further studies on the usefulness of quantitative DWI of small colonic lesions as a diagnostic and prognostic biomarker are highly relevant, including the addition of radiomics and artificial intelligence to DWI [22,23]. In colon tumors and adenomas, DWI offers the ability to non-invasively evaluate tissue cellularity. DWI enables clinicians to assess tumor density and extracellular characteristics. However, more data are needed to establish DWI as a robust and quantitative biomarker.

### Limitations

Our study population contained 18 pAdenomas and 5 pT1 tumors. We had 196 eligible patients from the endoscopic unit; some patients with small adenomas seen on endoscopic exams might not have been referred to MRI. This could cause lesion selection. This study provides a new potential method to evaluate pAdenomas and pT1 tumors, but more studies, with more patients, are needed to understand the potential of this method. It is important to recognize that the absolute ADC values are highly dependent on the scanner type, and variance in ADC must be considered when using ADC for diagnostic purposes. 

We had only one experienced observer. A previous inter-observer study showed a fair agreement for ADC measurements [27].

Using DWI to differentiate between malignant and benign findings is widely explored in other types of cancer, such as breast, prostate, and liver cancer. MRI of breast and prostate are less susceptible to blurring and movement artefacts from breathing and peristalsis compared to the colon. These advantages are especially apparent in small tumors, which can be difficult to detect on MRI images. While visible on T2 images, some colon tumors included in this study were difficult to measure on ADC images, potentially affecting the results.

Histopathology is an established gold standard. However, is this the best way, or are there other methods that have the potential to provide better tumor staging? For example, deep learning algorithms have the potential to play a role, either as a potential new gold standard or as an add-on. However, currently, their generalization is limited due to accepted validated datasets [28,29].

## 5. Conclusions

This study explored the utility of MRI DWI as a diagnostic biomarker for benign colon adenoma, early and advanced colon cancer, and for comparing mucinous-producing colon cancers. Quantitative DW-MRI is potentially useful for determining whether a colonic tumor is benign or malignant. We found a significant mADC mean difference between colon adenomas and early colon cancers, with an AUC of 0.80. Mucinous colon cancer shows less diffusion restriction when compared to non-mucinous colon cancer, a potential pitfall.

## Figures and Tables

**Table 1:** MRI sequence parameters. Abbreviations: DWI, diffusion-weighted imaging; NEX, number of excitations; TE, echo time; TR, repetition time. Angulated, perpendicular to the tumor.

	Voxel Size (mm)	Field of View (mm)	Matrix	NEX	TE/TR (ms)	Scan Time (min:s)
T2 Coronal	1.2 × 1.2 × 5	440 × 440 × 221	368 × 368	1	132/2725	2:30
T2 Sagittal	1.2 × 1.2 × 6	440 × 440 × 221	368 × 368	1	132/2725	1:50
DWI (b0-b800)	3 × 3 × 5	450 × 359 × 179	152 × 117	1	80/1658	2:30
T2 Angulated	0.8 × 0.8 × 3	325 × 325 × 104	408 × 408	1	120/4564	2:53
DWI (b0-b1000) Angulated	2.6 × 2.6 × 4	190 × 116 × 104	72 × 48	1	86/4640	2:50

**Table 2:** Tumor location and characteristics. Abbreviations: mADC, main tumor mean apparent diffusion coefficient in 10^−6^ mm^2^/s; mrNMCC, non-mucinous colon cancer determined using magnetic resonance imaging; mrMCC, mucinous colon cancer determined using magnetic resonance imaging; SD, standard deviation.

Tumor Location & Characteristics	Coecum	Ascendens	Transversum	Descendens	Sigmoid	Total	Mean mADC [SD]	Mean Tumor Size in cm [Range]
pAdenoma	4	3	4	2	5	18	1110 [243]	3.3 [1.4–10.0]
pT1–pT2	5	7	4	3	16	35	828 [243]	2.1 [0.8–5.5]
pT3–pT4	16	32	10	10	29	97	763 [218]	5.1 [0.6–19.0]
pN0	11	18	10	7	24	70	801 [236]	4.0 [0.8–19.0]
pN+	10	21	4	6	21	62	757 [206]	4.2 [0.6–18.0]
mrNMCC	16	33	13	13	42	117	762 [214]	3.9 [0.6–18]
mrMCC	5	6	1	0	3	15	925 [245]	5.6 [3.5–19]

**Table 3:** Overview of the mean mADC values, CI, and *p*-values. * Compared to the group directly above. *p* values obtained using independent sample *t*-tests. Abbreviations: *n*, number of patients; mADC, main tumor mean apparent diffusion coefficient; SD, standard deviation; CI, confidence interval; mrNMCC, non-mucinous colon cancer determined using magnetic resonance imaging; mrMCC, mucinous colon cancer determined using magnetic resonance imaging.

	*n*	Mean mADC [SD] (10^−6^ mm^2^/s)	Mean Difference [95% CI] * (10^−6^ mm^2^/s)	*p* Value *
mrNMCC	117	762 [214]		
mrMCC	15	925 [245]	163 [46–282]	0.007
pAdenoma	18	1110 [243]	185 [10–358]	0.039
pT1–pT2	35	828 [232]	−282 [−419–−144]	<0.001
pT3–pT4	97	763 [218]	−65 [−151–22]	0.142
pN0	70	801 [236]		
pN+	62	757 [206]	−44 [−121–33]	0.261

**Table 4:** Receiver operating characteristic analysis. Abbreviations: CI, confidence interval; AUC, area under the curve; mADC, main tumor mean apparent diffusion coefficient; vs., versus; mrNMCC, non-mucinous colon cancer determined using magnetic resonance imaging; mrMCC, mucinous colon cancer determined using magnetic resonance imaging.

	AUC [95% CI]	Optimal mADC Cutoff (10^−6^ mm^2^/s)	Sensitivity	Specificity	Accuracy
pAdenoma vs. pT1–pT2	0.80 [0.68–0.93]	1018	77%	78%	77%
pT1–pT2 vs. pT3–pT4	0.59 [0.47–0.70]	811	61%	51%	58%
pN0 vs. pN+	0.56 [0.46–0.66]	802	61%	53%	57%
mrNMCC vs. mrMCC	0.72 [0.57–0.87]	836	80%	70%	71%

## Data Availability

Data are contained within the article.
